# Modulatory effects of Nigella sativa l. oil on the hippocampus of dizocilpine-induced schizophrenia in BALB/c MICE

**DOI:** 10.1192/j.eurpsy.2023.661

**Published:** 2023-07-19

**Authors:** R. O. Folarin, O. Owoeye, A. Malomo

**Affiliations:** ^1^Anatomy, Olabisi Onabanjo University, Sagamu; ^2^Anatomy, University of Ibadan, Ibadan, Nigeria

## Abstract

**Introduction:**

Schizophrenia is a neuropsychiatric disorder characterised by positive, negative and cognitive behavioral symptoms. Despite years of research, the need for suitable therapy remains elusive. *Nigella sativa* oil (NSO) is a medicinal plant notable for its dietary, neuroprotective and anti-inflammatory properties. However, there is paucity of information on its neuroprotective potentials in schizophrenia.

**Objectives:**

This study was designed to investigate the modulatory effects of NSO on the hippocampus of dizocilpine-induced schizophrenia in mice.

**Methods:**

Sixty 14-weeks old male BALB/c mice (23-25g) were divided into five groups (n=12); control (normal saline, 1 mL/kg), NSO (1 mL/kg), dizocilpine-control (0.5 mg/kg) all for 7 days, while NSO (1 mL/kg for 7 days) + dizocilpine (0.5 mg/kg, for another 7 days) for preventive measure, and dizocilpine (0.5 mg/kg for 7 days) + NSO (1 mL/kg for another 7 days) for reversal. Dizocilpine and NSO were administered intraperitoneally and orally, respectively. Open field box was used for stereotypic popping. Animals were euthanised after behavioral studies, and harvested brains were weighed. Hippocampal glutamate was determined spectrophotometrically. Neuronal arrangement, sizes and densities were determined in perfused brain tissues using haematoxylin and eosin stain. Dendritic arborisations were assessed using Golgi stain. Metabotropic glutamate receptor-II (mGluR-2) and Glia Fibrilliary Acidic Protein (GFAP) were evaluated immunohistochemically. Data were analysed using descriptive statistics and ANOVA at α_0.05._

**Results:**

Stereotypic popping was observed in dizocilpine-control but not in the preventive and reversal NSO-treated animals. The NSO increased glutamate levels in the reversal (0.19±0.00 μM/μg tissue) but not in the preventive (0.18±0.00 μM/μg tissue) groups relative to dizocilpine-control (0.18±0.00 μM/μg tissue). Hippocampal neuronal density was significantly increased by dizocilpine (21.25±1.11neurons/100μm^2^) but modulated by NSO in the preventive (17.25±0.51 neurons/100μm^2^) and reversal groups (12.00±0.71 neurons/100μm^2^). Significant neuronal de-arborisation that occurred in the dizocilpine-control (989.90±253.9 μm^2^/2.5mm^2^area) was inhibited by NSO in the preventive (1678±370.90 μm^2^/2.5mm^2^area) and reversal (1639±314.80 μm^2^/2.5mm^2^area) treatments. Compared to dizocilpine-control (4219±127.3 ODU), NSO increased mGluR-2 expression in the preventive (4945±17.00 ODU) and reversal (4116±24.97 ODU) groups. The GFAP expression in NSO-treated animals relative to dizocilpine-control (5510±38.45 ODU) was significantly reduced in the preventive (4945±17.00 ODU) and reversal (4116±24.97 ODU) measures.

**Conclusions:**

*Nigella sativa* oil mitigated schizophrenic symptoms induced by dizocilpine in mice via modulation of hippocampal glutamate, metabotropic glutamate receptor–II upregulation, astrogliosis inhibition and neuroprotective mechanisms.

**Disclosure of Interest:**

None Declared

